# Clinical Results of Comprehensive Medication Management Services in Primary Care in Belo Horizonte

**DOI:** 10.3390/pharmacy7020058

**Published:** 2019-06-12

**Authors:** Carina de Morais Neves, Mariana Martins Gonzaga do Nascimento, Daniela Álvares Machado Silva, Djenane Ramalho-de-Oliveira

**Affiliations:** 1 Center for Pharmaceutical Care Studies, College of Pharmacy, Universidade Federal de Minas Gerais, Belo Horizonte, MG 31270-901, Brazil; carinamneves@gmail.com (C.d.M.N.); dalvaresms@gmail.com (D.Á.M.S.); 2 Department of Pharmaceutical Sciences, College of Pharmacy, Universidade Federal de Minas Gerais, Belo Horizonte, MG 31270-901, Brazil; marianamgn@yahoo.com.br

**Keywords:** Comprehensive Medication Management, pharmaceutical care, primary health care, chronic diseases, clinical results

## Abstract

The high prevalence of chronic diseases and use of multiple medications identified in Primary Health Care (PHC) suggest the need for the implementation of Comprehensive Medication Management (CMM) services. This study aimed to evaluate the clinical results of CMM services in a Brazilian PHC setting. A quasi-experimental study was performed with patients followed-up for two years (*n* = 90). Factors associated with the detection of four drug therapy problems (DTP) or more in the initial assessment were evaluated (univariate and multivariate analyses), as well as the clinical impact observed in laboratory parameters (HbA1c, Blood Pressure, LDL- and HDL-covariance analysis). A predominance of women (61.1%), a mean age of 65.5 years, and a prevalence of polypharmacy (87.8%)—use of five or more drugs—were observed. A total of 441 DTP was identified, 252 required interventions with the prescriber, 67.9% of which were accepted and 59.6% were solved. The main DTP were ‘non-adherence’ (28.1%), ‘need for additional drug therapy’ (21.8%), and ‘low dose’ (19.5%). Hypertension was positively associated with the identification of four DTP or more. A statistically significant reduction was detected in all assessed laboratory parameters (*p* < 0.05). CMM services contributed to the resolution of DTP and improved clinical outcomes.

## 1. Introduction

Primary Health Care (PHC) is the patient’s gateway to the public health system and has significant participation in the management of chronic diseases and prevention of adverse health outcomes through the integration of preventive and curative actions [1]. In Brazil, its organizational axis is the Family Health Strategy (eSF—Equipe Saúde da Família), which, in turn, is supported by the Family Health Support Team (NASF—Núcleo de Apoio à Saúde da Família). The NASF was established in 2008 by the Ministry of Health and consists of multidisciplinary teams (composed of pharmacists, nutritionists, physical therapists, social workers, and others) that provide support to the work of the eSF (composed mainly of a primary care physician and a nurse) [2]. The demands for a multidisciplinary approach in primary health care is increasing, since a large part of the population followed in this scenario has multiple health conditions, uses many medications, and is now composed of older adults, representing about 12.6% of the Brazilian population [3].

Patients treated in the PHC have a high prevalence of chronic diseases, such as diabetes mellitus (DM), hypertension, and dyslipidemia and consequently use multiple medications. Therefore, adequate drug therapy management becomes necessary, considering the complex drug therapy of this population. Thus, the provision of Comprehensive Medication Management (CMM) services by a competent clinical pharmacist inserted in the NASF can be an important strategy to enhance health outcomes and the operationalization of PHC [4].

CMM is a clinical service grounded in the theoretical framework of Pharmaceutical Care Practice that aims at the responsible provision of drug therapy to achieve tangible results that improve the quality of life of the patients [5]. Thus, in CMM services, the pharmacist checks whether the medicines used by the patient are the most appropriate, if they are effective, safe, and convenient for use in daily living in order to optimize their drug therapy. Thus, when he/she detects a drug therapy problem (DTP), defined as an event or circumstance linked to drug therapy that may actually or potentially interfere with the expected treatment results, pharmacists intervene with the patient or the team to solve it. It is worth emphasizing that patients are central to this practice and in partnership with the pharmacist and other professionals define the best way to reach their therapeutic goals [6].

Studies have shown that CMM has a considerable impact on the enhancement of the treatment of chronic diseases and contributes significantly to improve patients’ clinical results [7,8,9,10]. However, few studies have shown the clinical impact of a CMM service in the Brazilian PHC and provided directly by a NASF pharmacist. Therefore, this study aims to describe a CMM service in the primary care of the Brazilian city of Belo Horizonte (MG) and to evaluate its clinical impact.

## 2. Materials and Methods

This study has a quasi-experimental longitudinal design, with a single group of patients that received the CMM service. It should be emphasized that the World Health Organization encourages the evaluation of the impact of the implementation of services through a quasi-experimental design, and accordingly this study reflects this recommendation, which consists of a baseline and post-intervention evaluation (uncontrolled before–after study type) [11,12].

### 2.1. Setting

The CMM service in question was provided by a single pharmacist in two community primary care clinics of the municipality of Belo Horizonte, Minas Gerais. The populations assigned to these two units were at the time of the study 22,000 and 15,023 users and were attended by, respectively, six and four eSFs.

The pharmacist has a weekly workload of 40 h divided into 20 h for technical and managerial activities (e.g., medicines inventory control and coordination of the drug dispensing activities) and 20 weekly hours for patient care activities (provision of CMM services, health education, and scheduled meetings for discussion of cases referred to NASF professionals).

### 2.2. The Patient Care Process

CMM services as its theoretical underpinnings the practice of pharmaceutical care, whose patient care process involves the use of a rational decision-making process called Pharmacotherapy Workup (PW), as proposed by Cipolle et al. [13,14] Thus, the care process utilized in these services includes the pharmacist’s assessment of all of a patient’s drug therapy, including over-the-counter, supplements and prescribed medications, in order to identify, prevent, and resolve DTP that might be preventing the patient to achieve his or her goals of therapy. Care plans are developed for each medical condition and follow-up evaluations are carried out to determine actual outcomes.

### 2.3. Study Design and Data Collection

In order to assess the impact of the CMM service, the documentation of the CMM encounters were analyzed retrospectively for the total number of patients (*n* = 90) followed-up by the pharmacist at the clinics over the two-year period (February 2015–February 2017). The following data were collected: age (full years at the first visit), gender, place of care at the initial assessment (at home or at the health service unit), number of health problems, number of medications used (prescribed and non-prescribed), number of interventions (with the prescriber or the patient) and their acceptability (intervention with the physician—accepted or not accepted), number of DTP identified and resolved, as well as their types.

DTP were categorized according to the PW process meaning they were classified according to an evaluation of the indication, effectiveness, safety, and convenience of each medication the patient was taking [5,6]. DTP associated with the indication of a drug product include unnecessary drug therapy (DTP1) or the need for additional drug therapy (DTP2). DTP related to effectiveness include ineffective drug product (DTP3) or a dosage too low to be effective (DTP4). In the safety category, the patient can have an adverse drug reaction (DTP5) or a dosage too high (DTP6). After evaluating the first three categories, the pharmacist will assess the convenience of the drug product for that specific patient, which might lead to the identification of a DTP of non-adherence (DTP7).

The Charlson Comorbidity Index (CCI) [15] was calculated for each patient enrolled in the service and the following initial (detected at the first or second CMM visit) and final (detected at the last visit) laboratory parameters were collected: glycosylated hemoglobin (HbA1c, in percentage), systolic blood pressure (SBP, in mmHg), diastolic blood pressure (DBP, in mmHg), low-density lipoprotein-cholesterol (LDLc, in mg/dL) and high-density lipoprotein-cholesterol (HDLc, in mg/dL).

### 2.4. Study Variables and Data Analysis

The data were first analyzed descriptively, measuring the absolute and relative frequencies of the qualitative variables and the mean and standard deviation (SD) of the quantitative variables. Then, univariate and multivariate analyses were performed to identify factors associated with the identification of multiple DTP in the initial assessment (first and second consultations), and, therefore, patients that should be prioritized to receive CMM services. For such purpose, the variable “number of initial DTP” was dichotomized (0–3 DTP, ≥4 DTP) and the dependent variable, therefore, was defined as the identification of four DTP or more. The independent variables defined were: age (22–69; ≥70 years), gender, number of health problems (0–2; ≥3), number of medications used (0–7; ≥8), the presence of hypertension and DM. The quantitative variables (age, CCI, number of medications, and number of DTP) were dichotomized according to their median.

Univariate analyses were performed using the Pearson’s chi-square test or Fisher’s exact test when the expected value for one or more cells was equal to or less than five. Independent variables with *p* < 0.20 in the univariate analysis were included in the multivariate model calculated by logistic regression. A probability test was used to compare the models and the Hosmer–Lemeshow test was run to evaluate the model’s quality of fit. Univariate and multivariate analyses were based on the odds ratio (OR) and their respective 95% confidence intervals estimated by logistic regression. A level of statistical significance of 5% was adopted to identify the characteristics independently associated with the dependent variable.

The clinical impact of the CMM service was assessed by comparing the initial and final clinical/laboratory parameters (HbA1c, SBP, DBP, LDLc, and HDLc). Only patients with more than one visit (68 patients) were considered, and among these, only those with a measurement of the specific clinical parameter at the initial and final visit. First, the normal distribution of the clinical variables was verified with the Shapiro–Wilk test. As all variables had a normal distribution, the difference between their initial and final values was assessed through covariance analysis with repeated measures (ANCOVA) adjusted by the following covariates in their continuous format: CCI, age, number of health problems, and number of medicines. Repeated measure designs facilitate the measurement of the impact of the treatment on each subject rather than just comparing the means between two groups. In this case, each subject serves as their control and the variability between subjects can be isolated. Also, the ANCOVA analysis enables us to control the difference between the two measures by other covariates, increasing the test’s sensitivity. All statistical analyses were performed by the Stata^®^ statistical program, version 12.

### 2.5. Ethical Aspects

This study was approved by the ethics committee of the Federal University of Minas Gerais, Report No. 25780314.4.0000.0149. Anonymity of the participants and confidentiality of the information were guaranteed.

## 3. Results

A female majority (*n* = 55, 61.1%), with a mean age of 66.5 ± 13.4 years (minimum: 22, maximum: 89) was observed among the patients followed up, revealing a predominance of elderly individuals with 60 years or more (*n* = 68; 75.6%). Table 1 shows the demographic, health and medication use profile of the patients enrolled in the CMM service.

A total of 251 consultations was performed by the pharmacist (mean ± SD = 2.8 ± 1.6). In the initial assessment, most patients were using five or more medications (*n* = 79; 87.8%), setting a mean of 7.6 ± 2.7 medications (minimum: 2, maximum: 18). The patients had a mean of 3.23 ± 1.4 health problems (minimum: 1, maximum: 7), with a mean CCI of 4.1 ± 0.2 (minimum = 0; maximum = 12). The most prevalent diseases were hypertension (*n* = 74; 82.2%), dyslipidemia (*n* = 69; 76.6%) and diabetes (*n* = 58; 64.4%).

A total of 346 DTP were identified in the initial assessment (mean = 3.8 ± 2.4; minimum = 0; maximum = 10; median = 3.5), and 441 DTP in all consultations, and the most prevalent were related to: ‘non-adherence’ (DTP 7—*n* = 124; 28.1%); ‘need for additional drug therapy’ (DTP 2—*n* = 96; 21.8%); and ‘low dose’ (DTP 4—*n* = 86; 19.5%). Table 2 describes the number of stratified DTP by type.

The main drugs associated with ‘non-adherence’ DTP were aspirin (*n* = 13; 10.5%) and simvastatin (*n* = 12; 9.7%). The drug cholecalciferol—vitamin D (*n* = 14; 14.6%) was the main drug requiring inclusion in drug therapy (‘need for additional therapy’ DTP). Regarding ‘low-dose’ DTP, the main drug involved was NPH insulin (*n* = 32; 37.2%). Of the total DTP, 59.6% (*n* = 263) were resolved (*n* = 263), 57.1% (*n* = 252) required interventions with the prescriber for resolution, and 67.9% (*n* = 171) of these interventions were accepted. Also, 364 interventions were performed directly with the patient (interventions performed vs. 82.5% of total DTP).

Table 3 shows the results of the univariate and multivariate analyses, which indicate that the presence of hypertension was independently and positively associated with the dependent variable, which was the identification of four DTP or more in the initial assessment, in a statistically significant way (*p* = 0.031).

After multiple adjustments, a statistically significant difference was detected between the initial and final values of HbA1c, SBP, DBP, LDLc, and HDLc (Table 4).

## 4. Discussion

The elderly majority among the patients assisted (75.6%) tended to increase the complexity of the service described in this study. This population has a high number of chronic diseases, as a consequence uses the health system more frequently, and usually consists of multiple-medication users [16], as indicated by the high mean number of health problems (3.2 ± 1.4) and drugs used by patients enrolled in the CMM service (7.6 ± 2.7). These findings reinforce the need to develop drug therapy qualification strategies such as the implementation of CMM services since the use of drugs in the elderly is generally associated with the occurrence of a more significant number of DTP [17].

With demographic and epidemiological transition, chronic and degenerative diseases have begun to gain greater representativity [18]. This was reflected in the results of this study, where a high prevalence of hypertension, DM, and dyslipidemia was detected, with a high number of patients with three concomitant diseases (*n* = 44; 49.0%—a result not previously described). Such complexity evidences the required action of the clinical pharmacist who is in charge of the drug therapy of these patients.

Hypertension was the most prevalent (82.2%) chronic condition and was positively and independently associated with the identification of four or more DTP in the initial assessment. This result may be related to the fact that the PHC pharmacist has greater mastery and experience for decision-making and identification of DTP related to hypertension since it is a chronic condition of high national prevalence. Also, patients with cardiovascular diseases, such as hypertension, often prompt the clinical pharmacist to evaluate the presence of DTP related to indication or appropriateness of drug therapy that might lead to the inclusion of medications for adequate cardiovascular prevention [14]. This type of DTP was the second most frequently identified in the initial assessment of this study (22.3%—results not described).

In a study that evaluated the clinical, economic, and humanistic outcomes of CMM services provided over a ten-year period in the health system of Minnesota [19], the authors pointed out the need for additional medication (accounting for 28.1% of total DTP), the use of low dose (26.1%), and non-adherence to drug therapy (16.5%) as the most frequent DTP identified. This result is comparable to the DTP frequency of this study. However, the reversed incidence was observed among DTP. In the present study, the most frequent DTP was non-adherence (28.12%), followed by the need for additional drug therapy (21.77%) and lastly the low dose, representing 19.50% of the total DTP. Another study [20] with a sample of patients from South Australia also found that the most frequent DTP were non-adherence to drug therapy (31.7%), followed by the need of additional medication (15.9%), and then drug ineffectiveness (15.7%).

The leading causes of non-adherence DTP were forgetfulness and failing to understand the instructions, which is consistent with the study population of mostly elderly, and therefore being more susceptible to cognitive impairments. The high frequency of this type of DTP can contribute adversely to the clinical outcomes of this population since the understanding, acceptability, and continuity of medication use are necessary conditions for the adequate management of chronic diseases. Thus, interventions with the patient are essential in resolving these DTP and showed high frequency in this study (performed to solve 82.5% of DTP). It should be noted that this type of intervention is deeply rooted in the philosophy of pharmaceutical care practice, which aims for shared-decision making between pharmacists and patients and to empower patients to get closely involved in the management of their own care [13].

Patients’ non-adherence to drug therapy, especially among patients with chronic diseases and with more complex treatments remains a challenge for health services [2]. Considering preventive pharmacological measures, the difficulty of patients to understand the need for a medication may be the main reason that aspirin and simvastatin were the most frequent drugs involved in the non-adherence DTP. In Brazil, a study carried out in the city of Araucária-Paraná found that, among hypertensive patients, non-adherence can reach 60% of users, and among the factors that contribute most are schooling, failing to understand the disease, family history of cardiovascular disease, and the type of drug combination used [21]. In Santa Catarina, a study showed that, among users of lipid-lowering drugs, non-adherence increased in an average period of 19 months, after the onset of treatment [22]. Among diabetic patients, the low adherence to treatment was estimated at 50%, leading to poor glycemic control [23]. Furthermore, among the elderly, self-reported rates of 62.9% of non-adherence to treatment were found, and polypharmacy (use of five or more medications) is related to this problem [24].

The second most prevalent DTP, the need for additional drug therapy, reinforces the need for an expanded view of the pharmacist, who should focus not only on the drug therapy already in place but also on the screening and identification of untreated health conditions. The drug that was most frequently associated with this type of DTP, namely, Cholecalciferol (Vitamin D), is used against hypovitaminosis, prevalent in the world, that has an even more significant representativeness and risk among the elderly, who were the majority of the patients attended in this study [25,26].

For this reason, the Brazilian Society of Endocrinology and Metabology recommends supplementation for individuals who are at risk of vitamin D deficiency, such as the elderly, patients with osteoporosis, and those with a history of falls and fractures [25]. Thus, it is common practice for the pharmacist to request the screening exam in the initial CMM assessment, since a high percentage of the population is elderly. When a deficiency is detected, the propaedeutic procedure is to start therapy and communicate the eSF during the matrix-based strategy meetings to guarantee continuity of care.

The third most prevalent DTP was too low a dosage, due to a sub-therapeutic dose, incorrect administration, or use at inappropriate intervals, while NPH insulin was the primary drug associated with this DTP. Several factors are considered as impediments in the treatment of DM, influencing the achievement of patients’ therapeutic goals and increasing the frequency of DTP. This condition requires complex treatment and involves the need for self-monitoring and frequent adjustments of insulin dose in a large number of patients [27]. Besides, DM changes the life of the patient and is in continuous clinical progression. The patients’ daily routine and non-pharmacological measures also influence the effectiveness of insulin treatment. Thus, health professionals must be specifically trained to care for patients in insulin therapy, as the therapeutic goals should be individualized according to the characteristics of the patient and the stage of the disease [28].

In this study, most of the interventions were accepted by the prescribers (67.9%; *n* = 171), in a proportion higher than that found in CMM services provided to patients with COPD (55.2% of the accepted interventions) [8], with cancer (59.6%) [29], and with hypertension (42.7%) [30]. This may have influenced the high number of DTP resolved (59.6%). This result reflects the strengthening of the working relationship between the pharmacist and physicians, with a growing confidence in the pharmacist’s clinical competence due to her participation in the workflows of the healthcare units. By allowing discussions of patient cases directly with prescribers on a regular basis, the matrix-based strategies (within or outside the fixed schedule) seem to favor the reassessment of the therapeutic goals by the healthcare team and the shared construction of care plans. The necessary communication between professionals rarely occurred through letters or phone calls, which were used only when the pharmacist needed to contact professionals working outside her healthcare network.

A statistically significant decline was observed in all clinical and laboratory parameters (HbA1c, SBP, DBP, LDL, and HDL) of the CMM patients at the end of the follow-up period. A quasi-experimental longitudinal study conducted by Bunting (2008) [31] in Asheville also evaluated the clinical outcomes of a CMM service. The service was provided to patients with hypertension and dyslipidemia for six years, and the results also showed reduced SBP parameters (137.3–126.3 mmHg; *p* < 0.001); DBP (82.6–77.8 mmHg; *p* < 0.001); LDL (127.2–108.3 mg/dL; *p* < 0.001); TC (211.4–184.3 mg/dL; *p* < 0.001) and TG (192.8–154.4 mg/dL; *p* < 0.001).

Some limitations were found in this study. First, there was a single pharmacist providing CMM who was still a novice at the implementation stage of the service. The pharmacist was still in the process of learning the practice, the method of documentation, and all that is involved in the process of transformation from a traditional pharmacist to a pharmaceutical care practitioner. The lack of training and the fact that the service was provided by only one professional could have reduced the quality and completeness of the documentation and, in consequence, hindered the evaluation of the clinical impact in the present study. The process of documentation becomes perfected over time and requires professional mastery and experience and, as highlighted by Ramalho de Oliveira (2011), it directly affects the quality of the data collected, the care provided, and consequently the obtained and evaluated results [6].

Another limitation was the difficulty of the other members of the health team to understand the service provided, due to its non-institutionalization at the workplace pointed out by Machado-Silva et al. (2018) in a study developed in the same health care unit [32]. In the Brazilian context, the professional pharmacist acts with some constraints regarding drug therapy change when he/she deems it necessary, always depending on the prescriber to accept and implement the suggested changes [32,33]. Physicians generally do not expect pharmacists to have a clinical role in proposing interventions [32,33]. Also, professional turnover requires that physicians always be updated regarding the inclusion of a service that is new and, therefore, unknown, hampering the establishment of partnerships [32]. This may be one of the factors explaining interventions that were not accepted (*n* = 80; 31.8%).

We should also consider some factors such as the qualification of referrals. Many of the patients were referred to the pharmacist because of problems of non-adherence. The pharmacist could only make interventions or recommendations after receiving an update of the requested tests by the eSF, and this caused some delay in the establishment of clinical conducts. As reported by Cipolle (2012) [13], it is inconsistent to promote drug adherence without the evaluation of the indication, effectiveness, and safety parameters. Also, the CMM service requires that pharmacists be trained and updated in their clinical training to provide a quality and safe service for patients.

In addition, even though the present study demonstrated the significant impact that CMM services had in the improvement of clinical parameters of non-communicable diseases, the reduced number of patients limited these results to this particular setting and may not be applicable to a different patient population or other primary health care setting. However, we highlight the importance of studies such as the one hereby presented, since research describing and evaluating CMM services in the Brazilian PHC, which is the gateway for the largest universal public health care system, is still scarce.

## 5. Conclusions

This study showed that the CMM service had a significant impact on the clinical outcomes of patients. The clinical pharmacist was able to identify and propose interventions to solve and prevent DTP. As a result, the streamlining of drug therapy improved the control of the most prevalent chronic health conditions and proved to be a useful service in primary care. Thus, its incorporation into the PHC of the Brazilian health system should be considered a priority strategy for the control of prevalent chronic diseases.

## Figures and Tables

**Table 1:** Demographic, health and medication profile of patients enrolled in the Comprehensive Medication Management (CMM) service (*n* = 90). Belo Horizonte. 2015–2017.

Characteristic	*n* (%)
**Gender**	
Female	55 (61.1)
Male	35 (38.9)
**Age (full years)**	
22–68	50 (55.6)
≥69	40 (44.4)
Number of health problems in the initial assessment *	
0–2	28 (31.1)
≥3	62 (68.9)
**Charlson Comorbidity Index**	
0–3	41 (45.6)
≥2	49 (54.4)
Number of drugs in initial assessment *	
0–7	47 (52.2)
≥8	43 (47.8)
Number of DTP identified in initial visits *	
0–3	45 (50.0)
≥4	45 (50.0)

* Initial assessment = 1st and 2nd CMM visits.

**Table 2:** Absolute (*n*) and relative (%) frequency of drug-related problems (DTP) by category.

DTP Category	*n* (%)
1. Unnecessary medication	42 (9.5)
2. Need for additional drug therapy	96 (21.8)
3. Ineffective medication	19 (4.3)
4. Low dose	86 (19.5)
5. Adverse drug reaction	40 (9.1)
6. High dose	34 (7.7)
7. Non-adherence	124 (28.1)
Total	441 (100.0)

**Table 3:** Univariate and multivariate analysis of the characteristics associated with the dependent variable—identification of four or more drug-related problems in the initial assessment. Belo Horizonte. 2015–2017.

Variables	Univariate Analysis		Multivariate Analysis	
	OR (95% CI) **	*p*-Value ***	OR (95% CI) **	*p*-Value ****
**Age (full years)**				
22–69	1.0			
≥70	1.20 (0.52–2.75)	0.067	0.99 (0.41–2.44)	0.991
**Gender**				
Female	1.0			
Male	0.75 (0.32–1.77)	0.517		
**Number of health problems**				
0–2	1.0		1.00	
≥3	2.47 (1.16–5.29)	0.020	1.67 (0.63–4.41)	0.300
**Number of drugs**				
0–7	1.00		1.00	
≥8	1.88 (0.76–4.66)	0.172	2.27 (0.93–5.55)	0.071
**Hypertension**				
No	1.00		1.00	
Yes	5.69 (1.49–21.66)	0.006	4.56 (1.14–18.19)	0.031
**Diabetes Mellitus**				
No	1.00			
Yes	1.48 (0.62–3.52)	0.378		

Note: Number of DTP identified during the first and second visit; ** Odds ratio (95% CI) estimated by logistic regression; *** Pearson’s chi-square; included in the multivariate model when *p* < 0.20; **** Logistic regression; significant when *p* < 0.05.

**Table 4:** Comparison of initial and final values of defined clinical and laboratory parameters.

Parameter (Unit/Number of Evaluated Patients)	Median Initial Visit	Median Final Visit	*p*-Value **
HbA1c (%; *n* = 31)	8.4	7.8	<0.001
SBP (mmHg; *n* = 54)	136.5	132.2	0.020
DBP (mmHg; *n* = 54)	82.8	79.7	0.002
LDLc (mg/dL; *n* = 29)	119.7	109.1	<0.001
HDLc (mg/dL; *n* = 28)	45.3	50.4	<0.001

Note: HbA1c = glycosilated hemoglobin; SBP = Systolic blood pressure; DBP = Diastolic blood pressure; LDLc = Low-density lipoprotein cholesterol; HDLc = High-density lipoprotein cholesterol; ** Estimated by covariance analysis with repeated measures (ANCOVA) adjusted by the Charlson Comorbidity Index (ICC), age, number of health problems, and number of drugs.

## References

[1] Matta G.C., Morosini M.V.G. Atenção Primária à Saúde: Dicionário da Educação Profissional em Saúde, 2009, 23–28. http://www.midias.epsjv.fiocruz.br/upload/d/Atencao_Primaria_a_Saude_-_recortado.pdf.

[2] Brasil Ministério da Saúde. Secretaria de Atenção à Saúde. Departamento de Atenção Básica. Ferramentas para a Gestão e para o Trabalho Cotidiano: Núcleo de Apoio Saúde da Família. Brasília, 2014, 116p. http://bvsms.saude.gov.br/bvs/publicacoes/nucleo_apoio_saude_familia_cab39.pdf.

[3] IBGE Instituto Brasileiro de Geografia e Estatística: Síntese de Indicadores Sociais: Uma Análise das Condições de vida da População Brasileira, 2013. https://biblioteca.ibge.gov.br/visualizacao/livros/liv66777.pdf.

[4] Brasil Secretaria de Atenção à Saúde. Departamento de Atenção Básica. Práticas Farmacêuticas no Núcleo de Apoio à Saúde da Família (Nasf). Brasília: Ministério da Saúde, 2017. http://www.saude.goiania.go.gov.br/docs/divulgacao/NASF_praticas_farmaceuticas_nasf_2017.pdf.

[5] Hepler C.D., Strand L.M. (1990). Opportunities and responsibilities in pharmaceutical care. Am. J. Hospital. Pharm..

[6] Ramalho de Oliveira D. (2011). Atenção Farmacêutica: Da Filosofia ao Gerenciamento da Terapia Medicamentosa.

[7] Mendonça S.A.M., Melo A.C., Pereira G.C.C., Santos D.M.D.S.D., Grossi E.B., Sousa M.D.C.V.B., de Ramalho de Oliveira D., Soares A.C. (2016). Clinical outcomes of medication therapy management services in primary health care. Braz. J. Pharm. Sci..

[8] Detoni K.B., Oliveira I.V., Nascimento M.M., Caux T.R., Alves M.R., Ramalho-de-Oliveira D. (2016). Impact of a medication therapy management service on the clinical status of patients with chronic obstructive pulmonary disease. Int. J. Clin. Pharm..

[9] Obreli-neto P.R., Marusic S., Guidoni C.M., Baldoni Ade O., Renovato R.D., Pilger D., Cuman R.K., Pereira L.R. (2015). Economic evaluation of a pharmaceutical care program for elderly diabetic and hypertensive patients in primary health care: A 36-month randomized controlled clinical trial. J. Manag. Care Spec. Pharm..

[10] Cid A.S. (2008). Avaliação da Efetividade da Atenção Farmacêutica no Controle da Hipertensão Arterial. Master’s Thesis.

[11] Peter D.H., Tran N.T., Adam T. (2013). Implementation Research in Health: A Practical Guide.

[12] Grimshaw J., Campbell M., Eccles M., Steen N. (2000). Experimental and quasi-experimental designs for evaluating guideline implementation strategies. Fam. Pract..

[13] Cipolle R.J., Strand L.M., Morley P.C. (2012). Pharmaceutical Care Practice: The Patient Centered Approach to Medication Management.

[14] Cipolle R.J., Strand L.M., Morley P.C. (2004). Pharmaceutical Care Practice: The Clinician’s Guide.

[15] Charlson M.E., Pompei P., Ales K.L., MacKenzie C.R. (1987). A new method of classifying prognostic comorbidity in longitudinal studies: Development and validation. J. Chron. Dis..

[16] Veras R. (2009). Envelhecimento populacional contemporâneo: Demandas, desafios e inovações—Populationagingtoday: Demands, challengesandinnovations. Rev. Saúde Pública.

[17] Jansen P.A., Brouwers J.R. (2012). Clinical Pharmacology in Old Persons. Scientifica.

[18] Monteiro M. (2000). As transições demográfica e epidemiológica no Brasil. Acta Paul. Enferm..

[19] Isetts B.J., Schondelmeyer S.W., Artz M.B., Lenarz L.A., Heaton A.H., Wadd W.B., Brown L.M., Cipolle R.J. (2008). Clinical and economic outcomes of medication therapy management services: The Minnesota experience. J. Am. Pharm. Assoc..

[20] Rao D., Gilbert A., Strand L.M., Cipolle R.J. (2007). Drug therapy problems found in ambulatory patient populations in Minnesota and South Australia. Pharm. World Sci..

[21] Melchiors A.C. (2008). Hipertensão Arterial: Análise dos Fatores Relacionados com o Controle Pressórico e a Qualidade de Vida. Master’s Thesis.

[22] Cunico C. (2011). Dislipidemia e Efetividade do Uso de Hipolipemiantes em População do Extremo Oeste do Estado de Santa Catarina. Master’s Thesis.

[23] Souza R.A.P., Correr C., Melchiors A. (2011). Determinants of glycemic control and quality of life in type 2 diabetic patients. Lat. Am. J. Pharm..

[24] Rocha C.H. (2008). Medication adherence of elderly in Porto Alegre, RS. Ciênc Saúde Coletiva.

[25] Maeda S.S. (2014). Recomendações da Sociedade Brasileira de Endocrinologia e Metabologia (SBEM) para o diagnóstico e tratamento da hipovitaminose D. Arq. Bras. Endocrinol. Metab..

[26] Bandeira F., Griz L., Dreyer P., Eufrazino C., Bandeira C., Freese E. (2006). Vitamin D deficiency: A global perspective. Arq. Bras. Endocrinol. Metab..

[27] Sociedade Brasileira de Diabetes (2016). Diretrizes da Sociedade Brasileira de Diabetes (2015–2016).

[28] Chatterjee S., Davies M.J. (2015). Current management of diabetes mellitus and future directions in care. Postgrad. Med. J..

[29] Bremberg E.R., Hising C., Nylén U., Ehrsson H., Eksborg S. (2006). An evaluation of pharmacist contribution to an oncology ward in a Swedish hospital. J. Oncol. Pharm. Pract..

[30] Sookaneknum P., Richards R.M., Sanguansermsri J., Teerasut C. (2004). Pharmacist involvement in primary care improves hypertensive patient clinical outcomes. Ann. Pharmacother..

[31] Bunting B.A., Smith B.H., Sutherland S.E. (2008). The Asheville Project: Clinical and economic outcomes of a community-based long-term medication therapy management program for hypertension and dyslipidemia. J. Am. Pharm Assoc..

[32] Machado-Silva D.A., Medina S.D., Ramalho de Oliveira D. (2018). Clinical practice of pharmacists in family health Support team. Trab. Educ Saúde.

[33] Machado-Silva D.A., Mendonça S.A.M., O´Dougherty M., Ramalho de Oliveira D., Chemello C. (2017). Autoethnography as an Instrument for Professional (Trans) Formation in Pharmaceutical Care Practice. Qualit. Rep..

